# Metabolite-Sensing Receptors: Emerging Targets for Modulating Chronic Pain Pathways

**DOI:** 10.3390/cimb47010063

**Published:** 2025-01-17

**Authors:** Ciprian Pușcașu, Corina Andrei, Octavian Tudorel Olaru, Anca Zanfirescu

**Affiliations:** Faculty of Pharmacy, “Carol Davila” University of Medicine and Pharmacy, Traian Vuia 6, 020956 Bucharest, Romania; ciprian.puscasu@umfcd.ro (C.P.); octavian.olaru@umfcd.ro (O.T.O.); anca.zanfirescu@umfcd.ro (A.Z.)

**Keywords:** chronic pain, metabolites, long-chain fatty acids, inflammation

## Abstract

Chronic pain is a debilitating condition affecting millions worldwide, often resulting from complex interactions between the nervous and immune systems. Recent advances highlight the critical role of metabolite-sensing G protein-coupled receptors (GPCRs) in various chronic pain types. These receptors link metabolic changes with cellular responses, influencing inflammatory and degenerative processes. Receptors such as free fatty acid receptor 1 (FFAR1/GPR40), free fatty acid receptor 4 (FFAR4/GPR120), free fatty acid receptor 2 (FFAR2/GPR43), and Takeda G protein-coupled receptor 5 (TGR5/GPR131/GPBAR1) are key modulators of nociceptive signaling. GPR40, activated by long-chain fatty acids, exhibits strong anti-inflammatory effects by reducing cytokine expression. Butyrate-activated GPR43 inhibits inflammatory mediators like nitric oxide synthase-2 and cyclooxygenase-2, mitigating inflammation. TGR5, activated by bile acids, regulates inflammation and cellular senescence through pathways like NF-κB and p38. These receptors are promising therapeutic targets in chronic pain, addressing the metabolic and inflammatory factors underlying nociceptive sensitization and tissue degeneration. This review explores the molecular mechanisms of metabolite-sensing receptors in chronic pain, their therapeutic potential, and challenges in clinical application. By uncovering these mechanisms, metabolite-sensing receptors could lead to safer, more effective pain management strategies.

## 1. Introduction: An Overview of Chronic Pain

Pain is a leading reason for seeking medical care. Although conditions like heart attacks, strokes, infections, cancer, and diabetes result in higher mortality rates, chronic pain stands out as a significant source of global suffering and disability [1]. The International Association for the Study of Pain characterizes pain as a distressing sensory and emotional experience associated with, or similar to, actual or potential tissue damage [2]. Chronic pain is described as pain that persists beyond the normal tissue healing time, typically considered to be 3 months in the absence of other contributing factors [3].

Globally, chronic pain affects over 30% of individuals, with the adjusted prevalence in developing countries estimated at 18% [2,4]. In the United States, an estimated 20.4% of adults live with chronic pain, while 8% suffer from high-impact pain that significantly interferes with their daily lives [5]. Chronic pain also persists longer than many other conditions, with an incidence rate of 52.4 cases per 1000 person-years, surpassing diabetes, depression, and hypertension [6].

Chronic pain is a complex condition that arises from dynamic changes in the nervous system, distinguishing it from acute pain, which serves as a protective response to injury [7]. These changes involve profound plasticity in peripheral and central pain pathways, leading to the amplification and persistence of pain signals [8]. Furthermore, chronic pain is driven by complex interactions among neural, immune, and endocrine systems, with neuroinflammation and central sensitization playing key roles [9,10,11,12]. Processes such as glial cell activation further contribute to pain maintenance by releasing pro-inflammatory cytokines, perpetuating sensitization, and creating opportunities for novel therapeutic interventions [13].

Despite substantial advancements in understanding pain mechanisms, many treatments remain inadequate, with a focus on symptom management rather than addressing underlying causes. Conventional pharmacological therapies, such as opioids, non-steroidal anti-inflammatory drugs, and acetaminophen, often come with severe limitations, including addiction potential, side effects, and reduced efficacy over time. As such, there is an urgent need for novel therapeutic strategies targeting the root mechanisms of chronic pain [14,15,16,17,18,19].

Recent research has revealed the critical role of metabolic processes in chronic pain development and persistence. Dysregulated metabolic processes, including glycolysis, oxidative phosphorylation, and the tricarboxylic acid cycle, have been linked to neuroinflammation and heightened nociceptor sensitivity [20,21,22]. Key metabolites, such as lactate, succinate, bile acids, and reactive oxygen species, serve as signaling molecules that exacerbate neuroinflammatory responses, creating feedback loops that sustain chronic pain states [21,22,23,24].

Metabolite-sensing receptors, a subset of G protein-coupled receptors (GPCRs), have emerged as key regulators at the intersection of metabolic, immune, and nociceptive systems. GPCRs represent the largest family of membrane proteins in mammals, playing a pivotal role in diverse physiological processes, including nociception, metabolism, and immunity [25,26]. Recently, several of these receptors have been “deorphanized”, revealing their activation by key metabolic intermediates such as free fatty acids, succinate, and lactate [25]. These metabolite-sensing GPCRs act as molecular bridges between metabolic and immune systems, positioning them at the forefront of the emerging field of immunometabolism [27].

Specific metabolite-sensing GPCRs, such as GPR7 and GPR40, have been implicated in both the promotion and resolution of inflammation, making them critical regulators of pain pathways. GPR120 suppresses neuroinflammation by inhibiting pro-inflammatory pathways linked to tumor necrosis factor-alpha (TNF-α) and Toll-like receptor 4 (TLR4) signaling [28,29]. Similarly, GPR91 mediates responses to succinate, a metabolite that accumulates under metabolic stress, linking it to both immune activation and metabolic adaptation [30]. These receptors’ dual roles in modulating inflammation and pain highlight their potential as therapeutic targets for chronic pain management.

Despite the growing body of evidence, no comprehensive review currently exists to summarize the role of metabolite-sensing receptors in chronic pain pathways. Researchers and clinicians need an integrated overview of how these receptors contribute to chronic pain and how they can be targeted to develop effective therapies. By bridging knowledge gaps and highlighting recent advancements, this review explores the emerging roles of metabolite-sensing GPCRs in chronic pain pathways.

## 2. Metabolite-Sensing Receptors

### 2.1. General Signaling Mechanisms of GPCRs

Metabolite-sensing GPCRs bind to various metabolites and transmit signals that are important for proper immune and metabolic functions. Members of this family include GPR3, GPR7, GPR18, GPR40, GPR41, GPR109A, GPR120, GPR84, GPR35, and GPR91. In addition, bile acid receptors such as TGR5 (GPR131/GPBAR1) and proton-sensing receptors such as GPR65 show similar features [31].

GPCRs are integral membrane proteins that transduce extracellular signals into intracellular responses by interacting with various transducers, primarily G proteins and β-arrestins. The classical signaling mechanism involves the binding of a ligand to the extracellular domain of the GPCR, triggering a conformational change in the receptor. This conformational change activates the associated heterotrimeric G protein by facilitating the change in guanosine diphosphate to guanosine triphosphate on the Gα subunit, leading to the dissociation of Gα from the Gβγ dimer. These activated subunits then interact with downstream effectors such as adenylyl cyclase, phospholipase C, and ion channels, generating second messengers like cyclic AMP (cAMP) and inositol trisphosphate (IP3) [32].

Besides classical G protein signaling, GPCRs engage in β-arrestin-dependent pathways, which not only terminate G protein signaling but also initiate distinct downstream cascades. β-arrestins act as scaffolds to recruit signaling complexes, leading to the activation of mitogen-activated protein kinases (MAPKs) and other pathways. This bifurcation of signaling pathways, or biased signaling, enables GPCRs to mediate diverse physiological effects based on the ligand’s ability to preferentially stabilize specific receptor conformations [33].

Furthermore, following internalization, GPCRs continue to signal from endosomal membranes, sustaining G protein-mediated signaling. This compartmentalized signaling extends the temporal and spatial dynamics of the receptor’s activity, impacting physiological outcomes significantly [34]. Furthermore, phosphorylation patterns on the GPCR, termed phosphorylation “barcodes”, influence β-arrestin recruitment and downstream signaling specificity, adding a cell-type-specific dimension to receptor signaling [35]. Structural studies have highlighted the importance of conformational changes within the receptor’s transmembrane helices, particularly the outward movement of transmembrane helix 6 (TM6), in facilitating G protein activation [36].

The diversity of GPCR signaling is further enhanced by allosteric modulators, which bind to sites distinct from the orthosteric ligand-binding pocket, modulating receptor activity. Sodium ions, for example, act as allosteric modulators in class A GPCRs, influencing agonist binding and receptor activation [37].

Additionally, GPCR signaling regulates histone deacetylase (HDAC) activity through intracellular signaling and direct interactions. For example, GPCR activation via cAMP and protein kinase A (PKA) pathways modulates HDAC nuclear localization and activity, such as hypo-phosphorylation of HDAC5, which inhibits pathological MEF2 activation in cardiac cells [38]. GPCR kinase 5 (GRK5) phosphorylates HDAC6 to enhance its deacetylase activity, impacting resistance to chemotherapeutic agents such as paclitaxel in cancer cells [39]. GPCR-induced HDAC influences gene expression, chromatin remodeling, and cellular responses, linking extracellular signals to epigenetic regulation and opening potential therapeutic avenues for diseases involving aberrant HDAC activity [40].

In conclusion, the signaling mechanisms of GPCRs are highly dynamic and multifaceted, encompassing classical G protein signaling, β-arrestin-dependent pathways, and intracellular signaling. These mechanisms are regulated by receptor conformation, phosphorylation patterns, and allosteric modulators, allowing GPCRs to mediate diverse and context-specific physiological responses.

### 2.2. Structural Characteristics and Ligand Specificity of Metabolite-Sensing Receptors

Metabolite-sensing GPCRs represent a critical subset of the GPCR superfamily, detecting a wide range of endogenous ligands derived from metabolic processes. These receptors, such as GPR3, GPR7, GRP18, GPR40 (FFAR1), GPR120 (FFAR4), GPR160, and TGR5 (GPR131/GPBAR1), are integral in linking metabolic signals to physiological responses, including energy homeostasis, inflammation, and metabolic regulation [25].

GPR3 is highly expressed in the thyroid, adipose tissue, lung, and small intestine [41], exerting a diverse range of effects. In the central nervous system, it is expressed in various regions, including the cerebral cortex, striatum, amygdala, and hippocampus, where it regulates stress responses, anxiety, and despair-like behaviors. In cerebellar granular neurons, GPR3 promotes neuronal differentiation, synapsis formation, and nervous system development [42]. GPR3 receptors could play a role in promoting axonal regeneration in C-fibers, serving as a protective mechanism following nerve injury and contributing significantly to neural repair and growth [43].

GPR3 exhibits high constitutive activity due to its basal coupling with Gs proteins and its interaction with oleoylethanolamide (OEA). Even in the absence of OEA, GPR3 maintains basal activity by adopting a conformation between active and inactive states. When bound to OEA, this activity is further stabilized, enhancing GTP turnover and cAMP signaling. OEA binds to a hydrophobic tunnel in GPR3’s orthosteric pocket, formed by transmembrane helices TM3, TM5, TM6, and TM7. This binding is supported by key residues, such as phenylalanine at position 263, tryptophan at position 260, and tyrosine at position 280, along with water-mediated hydrogen bonding to OEA’s hydroxyethyl group. Mutations in these residues significantly impair receptor activity, underscoring their critical role [44].

GPR7 regulates feeding behavior [45], the release of pituitary hormones, and nociception [46]. It is primarily expressed in the central nervous system, in structures such as the spinal cord, hippocampus, thalamus, and amygdala [47,48,49]. Neuropeptides W (NPW) and B (NPB), known for their roles in energy regulation, are their endogenous ligands [50].

Co-administration of GPR7 agonists with morphine mitigates the side effects associated with the latter, such as conditioned place preference (indicative of reward behavior) and constipation, particularly at low doses [51]. Their synergistic analgesic effect has been attributed to the co-localization of μ-opioid receptors and NPBW1 receptors in the dorsal horn of the spinal cord and shared downstream signaling, such as enhanced ERK1/2 phosphorylation and cAMP modulation [51].

GPR18, also known as the N-arachidonyl glycine (NAGly) receptor, is a novel cannabinoid receptor activated by endocannabinoids such as anandamide (AEA). It interacts with Gαi/o, Gαq/11, and Gα12/13 proteins [52] and is expressed in lymphoid tissues, testis, ovary, lung, and brain regions, including the thalamus, cerebral cortex, hippocampus, and striatum [53]. GPR18 signaling facilitates neuron–microglia communication, influencing microglial migration, phenotype switching, and cytokine release. Endogenous ligands, NAGly and N-arachidonoyl serotonin, activate GPR18 by increasing intracellular Ca^2+^ levels [54]. In chronic neuropathic pain, impaired NAGly synthesis or degradation reduces GPR18 activation in the ventrolateral periaqueductal gray, disrupting descending pain control and enabling pain facilitation. Experimental activation of GPR18 by its ligands alleviated mechanical allodynia and thermal hyperalgesia in animal models of neuropathic pain without causing motor side effects [55], highlighting its therapeutic potential for pain management.

GPR40 is expressed in pancreatic β-cells, enhancing glucose-stimulated insulin secretion [56], as well as in macrophages [57], endothelial cells [58], adipocytes, and glial cells [59]. Its activation by long-chain fatty acids or selective agonists modulates multiple signaling pathways, initiating the Gαq signaling pathway characterized by phospholipase C-dependent synthesis of diacylglycerol (DAG) and IP3. This second messenger binds to its receptor located within the endoplasmic reticulum and induces the intracellular release of calcium ions. Contextually, DAG activates the protein kinase C that, in turn, initiates cytoskeleton remodeling necessary for cellular secretory activity. This also couples with Gαs and Gαi proteins, thus leading to a positive and negative modulation of cAMP levels, respectively [60].

GPR40 has two distinct binding sites: an orthosteric site within the transmembrane domain and an allosteric one near the lipid-rich region. The orthosteric site binds endogenous long-chain free fatty acids, such as palmitic acid, stabilizing the receptor through polar interactions with key residues in TM3, TM5, and TM7 to activate Gαq signaling and trigger calcium release [61]. The allosteric site, targeted by synthetic full agonists, stabilizes the receptor’s intracellular loop, enhancing G protein activation and enabling biased agonism through dual Gαq and Gαs signaling. This dual-site mechanism allows functional diversity, with the orthosteric site supporting baseline physiological functions like insulin secretion while the allosteric site amplifies signaling for therapeutic efficacy, making GPR40 a valuable target for conditions such as diabetes and pain management [59,62,63].

GPR43 has unique structural features and ligand specificity. It is primarily activated by short-chain fatty acids like acetate, propionate, and butyrate, which are produced during dietary fiber fermentation by gut microbiota. The receptor’s orthosteric binding pocket, located between transmembrane helices, facilitates ligand binding through hydrogen bonding and hydrophobic interactions. GPR43 couples with Gαi/o and Gαq/11 proteins, enabling the regulation of intracellular signaling pathways such as cAMP inhibition and calcium mobilization. These pathways contribute to its roles in modulating inflammatory responses, metabolic processes, and immune function [64,65].

In vitro studies highlight GPR43 as a potential target in osteoarthritic pain. In TNF-α-induced human fibroblast-like synoviocytes, activation of GPR43 significantly attenuated the inflammatory response, oxidative stress markers, and the expression of pro-inflammatory cytokines, chemokines, cellular adhesion molecules, and cartilage-degrading enzymes. Moreover, GPR43 activation inhibited NF-κB signaling by reducing p65 nuclear translocation [66]. In IL-1β-induced chondrocytes, butyrate-induced activation of GPR43 reduced the expression of pro-inflammatory enzymes such as nitric oxide synthase and cyclooxygenase, as well as inflammatory adipokines like lipocalin-2. It also inhibited adhesion molecules and key inflammatory pathways, including NF-κB and MAPK signaling [67].

GPR160 is upregulated in the spinal cord following traumatic nerve injury, contributing significantly to the development and persistence of neuropathic pain. The cocaine- and amphetamine-regulated transcript peptide (CARTp) functions as a ligand for GPR160. CARTp-GPR160 signaling induces hypersensitivity through ERK/CREB activation in the spinal cord [68].

TGR5 (GPR131/GPBAR1), activated by bile acids, is expressed in various tissues, including the liver, intestines, and immune cells. This receptor facilitates bile acid-mediated signaling, which impacts glucose metabolism, energy expenditure, and inflammation. The structural adaptability of TGR5’s binding pocket allows it to recognize diverse bile acid derivatives, positioning it as a crucial modulator in metabolic and immune responses [69]. Its orthosteric ligand-binding pocket is formed by TM2, TM3, TM5, and TM6, which stabilize bile acids and synthetic agonists through hydrogen bonding and hydrophobic interactions [70]. TGR5 activation triggers adenylate cyclase via Gs coupling, raising cAMP levels [71] and engaging MAPK and NF-κB signaling pathways depending on cellular context [72]. Structural changes, including outward movement of TM6 and elongation of TM5, facilitate G protein binding [70]. The gut flora–bile acid-TGR5/TRPV1 signaling pathway has been associated with diabetic peripheral neuropathic pain. Dysbiosis of gut flora impairs bile acid metabolism, reducing secondary bile acids and causing abnormal activation of the TGR5/TRPV1 pathway. Subsequently, peripheral nerve sensitization and pain occur [73].

## 3. The Role of Metabolite-Sensing Receptors in Chronic Pain

Preclinical studies indicate that the activation or inhibition of metabolite-sensing receptors influences various aspects of chronic pain, including inflammatory pain, neuropathic pain, and mechanical allodynia (Table 1), through mechanisms that often involve cytokine production, immune cell activation, and changes in neuronal excitability.

The GPR3 receptor selectively modulates thermal nociception via C-fibers but not mechanical nociception through Aβ-fibers. It supports C-fiber regeneration and neuroprotection, mitigating thermal pain sensitivity and enhancing morphine analgesia. However, GPR3 does not affect spinal microglial or astrocyte-driven inflammation [74].

The GPR7 receptor, expressed at low levels in healthy myelin-forming Schwann cells, is significantly upregulated in inflammatory neuropathies such as vasculitis and chronic inflammatory demyelinating polyradiculoneuropathy. This overexpression, restricted to myelin-forming Schwann cells, correlates with nociceptive signaling and inflammation [75]. Similarly, GPR7 expression, normally low in the spinal cord, is dramatically increased in inflammatory and immune-mediated neuropathies, as well as in animal models with immune-inflammatory or ligation-induced nerve injuries [75].

NPB/GPR7 signaling plays a key role in pain modulation. NPB-deficient mice demonstrated heightened sensitivity to inflammatory pain, exhibiting increased hyperalgesia during phase 1 of the formalin test and greater writhing behavior in the acetic acid test [76]. However, responses to non-inflammatory pain induced by magnesium sulfate were unaffected, underscoring the specific involvement of NPB/GPR7, but not NPW/GPR7, in modulating inflammatory pain pathways [76].

In neuropathic pain, GPR18 functions as a crucial receptor that responds to endogenous ligands, particularly NAGly. The activation of GPR18 has been shown to significantly mitigate mechanical allodynia and thermal hypersensitivity in animal models of neuropathic pain [55].

In pain modulation, GPR40 agonists, such as GW9508, demonstrated significant antinociceptive effects in neuropathic pain models (e.g., sciatic nerve ligation and formalin test). Intrathecal or brainstem injections reduced mechanical allodynia and thermal hyperalgesia, with the involvement of serotonergic and noradrenergic pathways. These effects, mediated by spinal IL-10 and β-endorphin, highlight GPR40’s role in descending pain control without motor side effects [59,77,78]. Additionally, oleic acid released by sensory neurons during nociceptive stimulation alleviated TRPV1-mediated thermal hypersensitivity via GPR40 and calcineurin-dependent mechanisms [79].

In inflammatory diseases, GPR40 showed protective roles. In a collagen-induced arthritis model, GPR40 deficiency exacerbated symptoms, leading to increased bone and cartilage damage through dysregulated B cell receptor (BCR) signaling. Reduced GPR40 expression in human RA patients correlated with disease severity, and agonist treatment mitigated excessive immune responses, positioning GPR40 as a therapeutic target in autoimmune disorders [80].

In diabetic neuropathy, GPR40 activation reversed vascular permeability, tight junction protein loss, and mechanical/thermal hypersensitivity in streptozotocin-induced diabetic mice, while its absence worsened nerve integrity [81]. Similarly, in a neonatal germinal matrix hemorrhage model, GPR40 activation improved neurological outcomes by promoting M2 microglial polarization and suppressing inflammation through PAK4/CREB/KDM6B signaling [82].

Lastly, GPR40 also enhances hypothalamic neurogenesis, as demonstrated in mice receiving intracerebroventricular GPR40 agonists. Treatment increased neural precursor proliferation, survival, and differentiation, mediated via p38 MAPK and BDNF signaling [83]. These findings collectively highlight GPR40’s therapeutic potential across neuropathic pain, neuroinflammation, autoimmune diseases, and neurodegeneration.

Activation of GPR43 has been implicated in the modulation of inflammation, primarily through the suppression of pro-inflammatory cytokine production and the modulation of immune cell function [64,66,67]. In animal models of colitis, the administration of GPR43 agonists has demonstrated significant therapeutic effects, including the reduction in inflammation and amelioration of clinical symptoms. Additionally, these agonists have been shown to reduce myeloperoxidase levels, a key biomarker of neutrophil infiltration and colonic inflammation [65].

Inhibiting GPR160 effectively alleviated both mechanical and cold hypersensitivity in chronic pain rodent models without affecting normal pain responses or inducing adverse effects. These results suggest selective alleviation of chronic pain states without impact on beneficial and protective nociceptive responses [68].

TGR5 plays a key role in chronic pain by alleviating mechanical allodynia and spontaneous pain, reducing neuroinflammation and glial activation, and promoting a shift from pro-inflammatory to anti-inflammatory macrophage phenotypes [71].

**Table 1:** Preclinical studies assessing the effectiveness of metabolite-sensing receptor modulation animal models of chronic pain.

Animal Model	Animal Strain	Treatment	Results	Author
Partial sciatic nerve ligation neuropathic pain model	Gpr3^−/−^ and Gpr3^+/+^ mice	-	Deletion of GPR3 receptor: ▪ increased sensitivity to thermal stimuli, both painful and non-painful, after nerve injury; ▪ did not impact mechanical sensitivity; ▪ reduced the effectiveness of morphine-induced analgesia without altering baseline pain thresholds	[74]
Partial sciatic nerve ligation	Sprague–Dawley rats	GPR7 agonists: neuropeptide W-23/B intrathecal injection 0.1–10 μg	Activation of GPR7: ▪ attenuated the level of mechanical allodynia in a dose-dependent manner	[84]
Formalin-induced pain Chronic constriction injury model	Sprague–Dawley rats	GPR7 agonists: neuropeptide W-23/B intrathecal injection 0.1–10 μg	Activation of GPR7: ▪ reduced acute inflammatory pain; ▪ reduced mechanical allodynia	[51]
Formalin-induced pain	Sprague–Dawley rats	GPR7 agonist NPW30 Microinjection 5 μg in the RVM, LC, and PAG	Activation of GPR7: ▪ reduced pain-related flinching behavior, particularly during the prolonged inflammatory phase (phase 2)	[85]
Partial ligation of the sciatic nerve neuropathic pain	Sprague–Dawley rats	N-arachidonyl-glycine intrathecal injections (700 nmol)	Activation of GPR18: ▪ significantly increased mechanical paw withdrawal threshold, reducing mechanical allodynia	[86]
Partial ligation of the sciatic nerve neuropathic pain	Sprague–Dawley rats	N-arachidonyl-glycine intrathecal injections (500 nmol bilaterally) into the ventrolateral PAG	Activation of GPR18: ▪ reduced mechanical allodynia; ▪ reduced thermal hypersensitivity	[55]
		siRNA 10^7^ IU into the ventrolateral PAG	Knockdown of GPR18: ▪ reduced GPR18 receptor mRNA; ▪ reduced analgesic effect of NAGly	
Complete Freund’s adjuvant-induced inflammatory chronic pain	Male ddY mice	GPR40 agonists: DHA (50 µg), GW9508 (1.0–25 µg) intracerebroventricular injection	Activation of GPR40: ▪ reduced pain responses; ▪ increased proopiomelanocortin-expressing neurons; ▪ increased c-Fos activation in the arcuate nucleus	[87]
Spinal nerve ligation neuropathic pain model Complete Freund’s adjuvant-induced inflammatory chronic pain Carrageenan-induced inflammatory pain	C57BL/6 J mice	GPR40 agonist GW950 intrathecal injection 100 pm	GPR40 activation dose-dependently: ▪ reduced ipsilateral mechanical allodynia and thermal hyperalgesia in neuropathic and inflammatory pain; ▪ decreased the frequency of spontaneous excitatory postsynaptic currents in the substantia gelatinosa neurons	[77]
L5/L6 spinal nerve ligation neuropathic pain model	Wistar rats	GPR40 agonist GW9508 intracerebroventricular injection 1 μg	Activation of GPR40: ▪ greatly reduced mechanical and thermal pain ▪ stimulated the expression of interleukin-10 (IL-10) and β-endorphin in spinal microglia and astrocytes	[59]
Formalin-induced pain	ddY mice	GPR40 agonist GW9508 intracerebroventricular injection 1 μg	Activation of GPR40: ▪ decreased formalin-induced pain behaviors, particularly in the late phase of the formalin test	[78]
Streptozotocin-induced type 1 diabetes mouse model	C57BL/6 mice	GPR40 agonist GW9508 (50 mg/kg)	Activation of GPR40: ▪ mitigated mechanical and thermal hypersensitivities associated with diabetic neuropathy	[81]
	GPR40 −/− mice	-	Deletion of GPR40: ▪ did not induce thermal hypersensitivity associated with diabetic neuropathy; ▪ induced mechanical hypersensitivity associated with diabetic neuropathy	
Zymosan-induced thermal hypersensitivity	C57BL/6NRj mice	GPR 40 agonist oleic acid 50 mM intraplantar injection	Activation of GPR40: ▪ alleviated thermal hypersensitivity	[79]
	GPR40 deficient mice	-	Knockout of GPR40: ▪ stimulated thermal hypersensitivity	
Formalin-induced pain	CD-1 mice	GPR 40 agonist quercetin-3-oleate 100 µg subcutaneous injection	Activation of GPR40: ▪ reduced nociceptive behavior in the formalin test; ▪ protected macrophages against a possible increase in reactive oxygen species; ▪ strongly and transiently increase intracellular calcium levels	[88]
Dextran sulfate sodium-induced colitis model	C57BL/6 mice	GPR43 agonists compounds 110 and 187 (30 mg/kg)	Activation of GPR43: ▪ reduced inflammation and irritation in the colon; ▪ restoration of colon length; ▪ decreasing myeloperoxidase expression	[65]
Chronic constriction injury model Spared nerve injury model	Rats (strain not specified)	siGpr160 or neutralizing anti-GPR160 Ab daily intrathecal injections	Activation of GPR160: ▪ reversed mechano and cold-allodynia; ▪ did not modify normal nociceptive thresholds	[68]
Sciatic nerve constriction neuropathic pain model	GPR160-floxed and GPR160 knockout mice	-	Knockout of GPR160: ▪ did not induce mechanical and cold allodynia	[89]
Spared nerve injury model	C57BL/6 mice	INT-777 5 μL intrathecal injection	Activation of TGR5: ▪ alleviated mechanical allodynia in a dose-dependent manner. Repeated administration led to sustained relief ▪ reduced activation of microglia and astrocytes and neuronal sensitization	[90]
Partial sciatic nerve ligation mouse model	C57BL/6J mice	INT-777 administered perisciatically at 50 mg/mL	Activation of TGR5: ▪ significantly alleviated mechanical allodynia and spontaneous pain; ▪ reduced neuroinflammation by decreasing pro-inflammatory mediators, such as CCL3, CXCL9, IL-6, and TNF-α; ▪ promoted a shift from pro-inflammatory (CD86^+^CD206^−^) to anti-inflammatory (CD86^−^CD206^+^) macrophages	[91]
		Cre-dependent shRNA lentivirus administered perineurally at 4 × 10^6^ TU	Knockdown of TGR5: ▪ increased mechanical allodynia and spontaneous pain ▪ increased expression of pro-inflammatory mediators, such as CCL3, IL-6, and TNF-α	

Legend: Ab, antibody; CCL3, chemokine (C-C motif) ligand 3; CXCL9, chemokine (C-X-C motif) ligand 9; DHA, docosahexaenoic acid; GPR, G-protein-coupled receptor; IL, interleukin; NAGly, N-arachidonyl-glycine; NPW30, neuropeptide W-30; PAG, periaqueductal gray; RVM, rostral ventromedial medulla; siGpr160, small interfering RNA targeting GPR160; TGR5, G-protein-coupled bile acid receptor 5; TNF-α, tumor necrosis factor-alpha.

## 4. Discussion

The exploration of metabolite-sensing receptors such as GPR3, GPR7, GPR18, GPR40, GPR160, and TGR5 highlights their immense potential as therapeutic targets in chronic pain. Each receptor demonstrates distinct mechanisms and effects on pain modulation, neuroinflammation, and neuronal plasticity, underscoring the versatility of this receptor family in addressing complex pathophysiological processes.

The evidence suggests that GPR3 offers a unique avenue for targeting thermal nociception without influencing mechanical sensitivity, positioning it as a potential adjunct therapy to enhance opioid efficacy while avoiding opioid-associated side effects [74]. However, its limited impact on spinal inflammation may reduce its utility in cases where neuroinflammatory mechanisms dominate chronic pain etiology.

Conversely, GPR7 emerges as a key player in inflammatory neuropathies and nociceptive signaling, with its upregulation in response to injury and inflammation. The specific role of NPB/GPR7 signaling in inflammatory pain, but not non-inflammatory pain, adds to its appeal as a targeted therapy for conditions characterized by heightened inflammatory responses [51,84,85]. However, the receptor’s limited specificity for mechanical pain pathways could pose challenges in addressing diverse chronic pain presentations.

GPR18 demonstrates significant potential in neuropathic pain by leveraging its role in neuron–microglia communication and its ability to alleviate mechanical allodynia and thermal hyperalgesia. Its cannabinoid-related signaling pathways offer a promising foundation for the development of non-opioid pain therapeutics, although its dependence on endogenous ligands like NAGly, whose synthesis may be impaired in chronic pain states, warrants further exploration [86].

GPR40 stands out for its multifaceted roles in metabolic, inflammatory, and nociceptive processes. The receptor’s engagement in neuroimmune pathways, such as IL-10 and β-endorphin signaling, further enhances its appeal [59,77,78,87]. However, the broad expression of GPR40 across various tissues necessitates caution to minimize off-target effects.

GPR160 and TGR5 also demonstrate promise, particularly in reducing hypersensitivity and neuroinflammation. TGR5’s ability to modulate macrophage polarization and its role in diabetic neuropathic pain underscore its importance in immune-mediated pain conditions [92]. However, challenges remain in translating preclinical findings into clinical success, particularly in identifying appropriate agonists or inhibitors with favorable safety and pharmacokinetic profiles.

**Figure 1:**
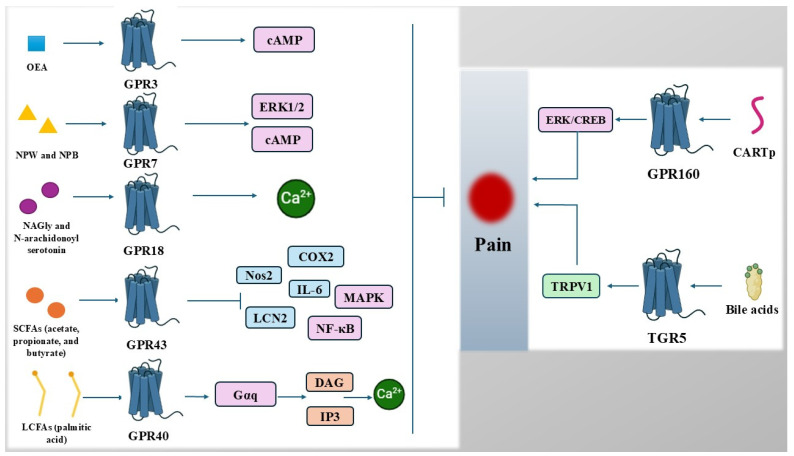
Diverse mechanisms of action of metabolite-sensing receptors in chronic pain Legend: CARTp, cocaine- and amphetamine-regulated transcript peptide; Ca^2+^, calcium; cAMP, cyclic adenosine monophosphate; CREB, cAMP Response Element-Binding Protein; DAG, diacylglycerol; ERK, extracellular signal-regulated kinase; GPR, G protein-coupled receptors; IL-6, interleukin 6; LCN2, lipocalin-2; LCFAs, long-chain fatty acids; NAGly, N-arachidonyl glycine; NF-κB, nuclear factor kappa B; NPB, neuropeptide B; NPW, neuropeptide W; Nos2, nitric oxide synthase 2; OEA, oleoylethanolamide; SCFAs, short-chain fatty acids; TRPV1, transient receptor potential vanilloid 1.

Collectively, the data emphasize the critical role of metabolite-sensing receptors in chronic pain and highlight their diverse mechanisms of action (Figure 1). These receptors not only bridge metabolic and immune pathways but also influence neuronal excitability and pain transmission, providing a multifaceted approach to pain management. Unlike traditional symptom-focused pain therapies, metabolite-sensing receptors target underlying mechanisms such as neuroinflammation, immune dysregulation, and metabolic dysfunction, providing a rational basis for therapeutic development [59,77,78,87]. Nevertheless, future research must address gaps such as ligand specificity, potential off-target effects, and the integration of these therapies into existing treatment paradigms [92]. By refining our understanding of these receptors, we can unlock novel and targeted strategies to alleviate chronic pain and improve patient outcomes.

While the presented data on metabolite-sensing receptors as therapeutic targets in chronic pain offer significant promise, several limitations and gaps in current evidence must be acknowledged. The evidence is derived from animal models, such as mice and rats, using specific pain-inducing paradigms like sciatic nerve ligation, formalin injection, and inflammatory agents [59,77,78,87].

Although these models mimic certain aspects of chronic pain, they may not fully capture the complexity and heterogeneity of human chronic pain conditions. Species differences in receptor expression, ligand specificity, and signaling pathways may limit the translational relevance of preclinical findings. For instance, receptor activity in rodents may not entirely reflect that in humans, particularly in terms of ligand–receptor binding affinities and downstream effects.

While the mechanistic pathways for receptors like GPR3 [74], GPR7 [51,84,85], GPR18 [86], and GPR40 [59,77,78] have been elucidated in part, gaps remain in fully understanding their molecular mechanisms. For example, the interplay between receptor activation and central sensitization in chronic pain states remains underexplored.

The complexity of chronic pain, influenced by factors like genetics, comorbidities, and psychosocial elements [93], may complicate the translation of receptor-targeted therapies into effective treatments for diverse patient populations. Current studies primarily focus on inflammatory and neuropathic pain models, with limited exploration of other chronic pain conditions, such as cancer pain or fibromyalgia.

Clinical investigations are currently being conducted to assess therapeutic agents that specifically target metabolite-sensing GPCRs in the management of various diseases. For example, GPR40 agonists such as TAK-875 [94], LY2881835 [95], P11187 [96], SHR0534 [97], and JTT-851 [98] have been evaluated in clinical trials for the treatment of type 2 diabetes. While certain compounds, including TAK-875, demonstrated initial efficacy, their development was subsequently discontinued due to concerns regarding hepatic toxicity [99]. However, there is a notable absence of clinical studies investigating medications that target metabolite-sensing GPCRs for the treatment of chronic pain. This gap in research underscores the need for future investigations to assess the efficacy and safety of such therapeutic agents in managing chronic pain conditions. Moreover, further research is needed to assess the potential contribution of mutations in these receptor genes to the pathophysiology of chronic pain conditions.

## 5. Conclusions

In conclusion, metabolite-sensing receptors such as GPR3, GPR7, GPR18, GPR40, GPR43, GPR160, and TGR5 represent promising therapeutic targets for chronic pain due to their ability to modulate neuroinflammation, nociception, and immune responses. These receptors bridge metabolic and immune systems, offering innovative approaches to address underlying mechanisms of pain rather than merely managing symptoms. Despite their potential, further research is needed to overcome translational challenges, refine ligand specificity, and ensure clinical applicability across diverse chronic pain conditions. Unlocking the therapeutic potential of these receptors could revolutionize chronic pain management and improve patient outcomes.

## Data Availability

All data generated or analyzed during this study are included in this published article.
